# Granular Swollen Epithelial Cells in Native Kidney Biopsies: Prevalence, Clinicopathological Associations and Kidney Outcomes

**DOI:** 10.3390/diagnostics16172822

**Published:** 2026-09-02

**Authors:** Naruhiko Uchida, Kenji Tsuji, Hiroyuki Nakanoh, Kazuhiko Fukushima, Shinji Kitamura, Jun Wada

**Affiliations:** Department of Nephrology, Rheumatology, Endocrinology and Metabolism, Okayama University Graduate School of Medicine, Dentistry and Pharmaceutical Sciences, 2-5-1 Shikata-cho, Okayama 700-8558, Japan

**Keywords:** granular swollen epithelial cells, native kidney biopsy, kidney outcomes, renal medulla, mitochondria

## Abstract

**Background/Objectives**: Granular swollen epithelial cells (GSECs) have been described primarily in mitochondrial cytopathies, in which they are regarded as a characteristic pathological finding that may aid in the diagnosis of mitochondrial dysfunction; however, their prevalence and clinical significance in native kidney disease remain unclear. We aimed to evaluate the prevalence, clinicopathological associations, and prognostic significance of GSECs in native kidney biopsy specimens. **Methods**: Among 1165 adults who underwent native kidney biopsy between 2011 and 2024, 501 patients with evaluable medullary tissue were included in this retrospective cohort study. GSECs were defined as enlarged tubular epithelial cells with conspicuous granular cytoplasm in medullary tubules, and cases were classified as GSEC-positive when at least one unequivocal GSEC was identified. Clinical and histopathological characteristics were compared between groups, and kidney outcomes were evaluated using Cox proportional hazards models. **Results**: The mean baseline eGFR was 60.8 ± 26.3 mL/min/1.73 m^2^, and the median urinary protein excretion was 1.1 g/gCr. During a median follow-up of 2.3 years, 112 patients (22.3%) reached the composite kidney outcome. GSECs were identified in 18% of native kidney biopsy specimens containing evaluable medullary tissue, and were observed across a broad spectrum of kidney diseases. GSEC positivity was associated with older age and underlying pathological diagnoses but was not associated with baseline kidney function or proteinuria. Kaplan–Meier and multivariable Cox analyses revealed no significant association between GSECs and kidney prognosis. **Conclusions**: GSECs are not specific to kidney allografts and are observed in a subset of diverse native kidney diseases. Although associated with certain clinical characteristics, no independent association between GSECs and kidney outcomes was observed in this cohort. These findings suggest that GSECs may reflect disease-dependent biological responses to tubular epithelial stress, potentially related to metabolic or mitochondrial alterations, rather than a marker of progressive kidney injury.

## 1. Introduction

Chronic kidney disease (CKD) represents a major global health burden, and identification of pathological and prognostic factors associated with kidney disease progression remains an important challenge in nephrology [1,2]. CKD arises from a wide range of underlying disorders, including immune-mediated glomerular diseases, diabetic kidney disease, hypertensive nephrosclerosis, and hereditary kidney diseases, each characterized by distinct pathological mechanisms and clinical courses [3,4]. Kidney biopsy is a fundamental tool for the diagnosis, prognostic assessment, and pathophysiological understanding of kidney diseases, providing detailed information on glomerular, tubulointerstitial, and vascular compartments [5,6]. Histopathological evaluation plays a central role not only in establishing a definitive diagnosis but also in guiding treatment decisions and predicting clinical outcomes [7,8]. For example, interstitial fibrosis and tubular atrophy (IFTA) are well established as key determinants of kidney prognosis across a wide range of diseases [9,10]. In contrast, certain morphological findings observed in tubular epithelial cells remain less well characterized, and their clinical and biological significance has not been fully elucidated [11]. The renal medulla, in particular, represents a unique microenvironment characterized by relatively low oxygen tension and high metabolic demand, rendering tubular epithelial cells especially susceptible to hypoxia and metabolic stress [12]. Morphological alterations observed in this compartment may therefore provide important insights into cellular adaptation, injury responses, and metabolic dysregulation [11].

Granular swollen epithelial cells (GSECs) are a distinctive histopathological finding characterized by enlarged tubular epithelial cells with conspicuous granular cytoplasm [13,,14]. GSECs were originally described in kidney biopsy specimens from patients with mitochondrial disease-associated renal involvement and are considered a histological feature reflecting mitochondrial dysfunction [15]. In these conditions, evaluation typically focuses on distal tubular epithelial cells and collecting duct epithelial cells, because proximal tubular epithelial cells are intrinsically rich in cytoplasmic granules and may be difficult to distinguish from GSEC-like changes. A previous study has reported that GSECs are present in around 20–30% of patients with mitochondrial diseases, indicating relatively low sensitivity despite their association with mitochondrial pathology [14]. Furthermore, GSECs are not specific to mitochondrial disorders and may also be observed in other clinical contexts [16,17]. Recently, similar morphological findings have been reported in kidney allografts, where their prevalence is lower, and their presence has been associated with aging-related changes and chronic injury [17]. Taken together, these observations suggest that GSECs may represent a morphological manifestation of altered cellular metabolism or mitochondrial dysfunction, rather than a disease-specific lesion.

Despite these insights, the clinical relevance of GSECs in native kidney disease remains unclear. Although GSECs are occasionally observed in routine native kidney biopsies, their pathological significance has not been systematically evaluated. In particular, it remains uncertain whether GSECs represent disease-specific lesions, markers of ongoing tubular injury, or non-specific morphological responses to cellular stress. Moreover, whether the presence of GSECs provides independent prognostic information for kidney outcomes in CKD patients has not been established. To address these gaps, we investigated the prevalence, clinicopathological associations, and prognostic significance of GSECs in a cohort of patients undergoing native kidney biopsy. We hypothesized that GSECs represent a nonspecific morphological manifestation of tubular epithelial stress rather than a disease-specific or prognostic lesion. To examine this hypothesis, we analyzed detailed clinical and histopathological data and evaluated kidney outcomes using multivariable models.

## 2. Materials and Methods

### 2.1. Study Design and Participants

**Figure 1:**
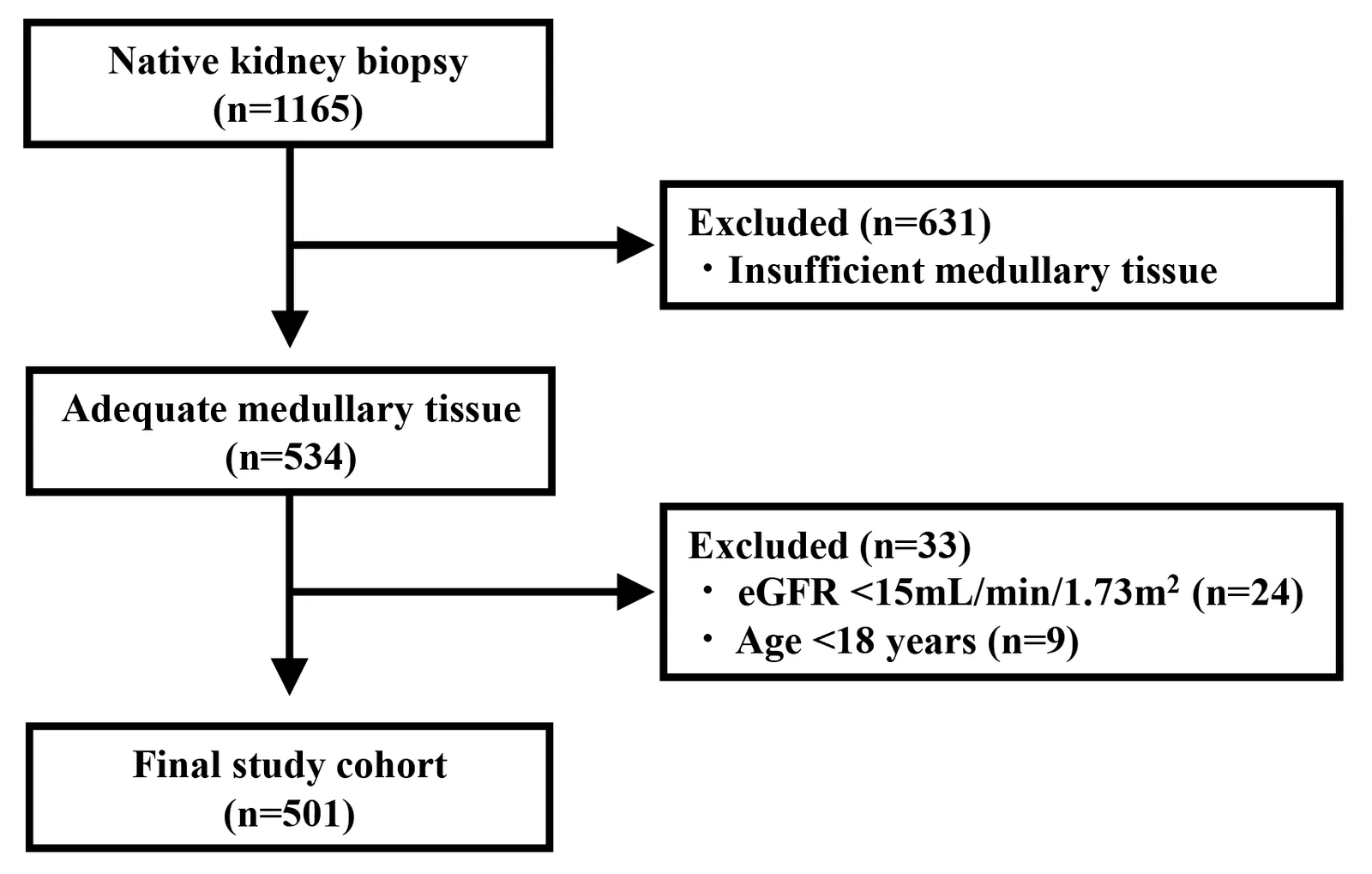
Flowchart of study participants.

We conducted a retrospective cohort study of adult patients who underwent native kidney biopsy at Okayama University Hospital (Okayama, Japan) between January 2011 and March 2024. During this period, a total of 1165 patients were screened. Patients were excluded if they had an estimated glomerular filtration rate (eGFR) ≤15 mL/min/1.73 m^2^, were receiving maintenance dialysis at the time of biopsy, were younger than 18 years of age, or had insufficient medullary tissue for evaluation. After applying these criteria, 501 patients were included in the final analysis (Figure 1). This study represents an expanded cohort that includes additional cases beyond those used in our previous analyses [18]. Baseline clinical data were collected at the time of kidney biopsy, and follow-up data were obtained from electronic medical records. The study protocol was approved by the Institutional Review Board of Okayama University Hospital (approval numbers: 1908-022 and 2407-038) and was conducted in accordance with the Declaration of Helsinki. Given the retrospective nature of the study, the requirement for written informed consent was waived; instead, information regarding the study was disclosed on the institutional website, and patients were given the opportunity to opt out. This study was reported in accordance with the Strengthening the Reporting of Observational Studies in Epidemiology (STROBE) guidelines (File S1).

### 2.2. Histological Parameters

Kidney biopsy specimens were processed for light microscopy, immunofluorescence, and electron microscopy according to standard protocols. For light microscopy, sections were stained with hematoxylin and eosin, periodic acid–Schiff, periodic acid–methenamine silver, and Masson’s trichrome. All biopsy specimens were independently reviewed by at least two experienced nephrologists who were blinded to clinical outcomes, and discrepancies were resolved by consensus at multidisciplinary conferences incorporating both clinical and pathological information. Tubulointerstitial lesions were evaluated semi-quantitatively. Cortical IFTA and inflammatory cell infiltration were graded on a scale of 0–3 (≤10%, 11–25%, 26–50%, and >50%), in accordance with established criteria including the Banff classification [19]. In the medulla, medullary fibrosis and cast formation were scored as 0 (<10%), 1 (10–24%), or 2 (≥25%), whereas medullary inflammatory cell infiltration was scored as 0 (<5%), 1 (5–14%), or 2 (≥15%), as previously reported [18].

### 2.3. Definition of GSECs

GSECs were defined based on previous reports as tubular epithelial cells exhibiting conspicuous cytoplasmic granularity and enlargement relative to adjacent tubular epithelial cells [15,17]. In the present study, GSECs were operationally defined as tubular epithelial cells showing prominent cytoplasmic granularity and apparent enlargement, with an approximately ≥1.5-fold increase in cell area compared with adjacent tubular epithelial cells. The approximately ≥1.5-fold increase in cell area was visually estimated by comparison with adjacent tubular epithelial cells and was not determined by formal morphometric analysis. GSECs were specifically evaluated in medullary tubules because of the distinct metabolic and hypoxic microenvironment of this compartment. Cases were classified as GSEC-positive when at least one unequivocal GSEC was identified in the examined sections. Because the primary objective was to evaluate the prevalence and clinical significance of GSECs, cases were classified dichotomously as positive or negative. Semi-quantitative assessment of GSEC burden was not performed and should be evaluated in future studies. Histopathological assessment of GSECs was independently performed by at least two nephrologists experienced in renal pathology who were blinded to the clinical diagnosis and clinical data at the time of evaluation. Cases with discrepant interpretations were subsequently reviewed jointly and classified by consensus discussion. Because enlarged eosinophilic tubular epithelial cells may occasionally overlap morphologically with reactive tubular changes or intercalated cells, evaluation was performed based on the combined presence of cellular enlargement and conspicuous granular cytoplasmic change.

### 2.4. Clinical Variables

Clinical and laboratory data at the time of biopsy were extracted from electronic medical records. These included age, sex, body mass index (BMI), hemoglobin A1c (HbA1c), use of angiotensin-converting enzyme inhibitors (ACE-Is) and/or angiotensin II receptor blockers (ARBs), systolic and diastolic blood pressure, smoking history, serum creatinine, eGFR, urinary protein excretion, and the presence of hematuria (>5 erythrocytes per high-power field). eGFR was calculated using the equation developed by the Japanese Society of Nephrology [20]. Hypertension was defined as a blood pressure ≥140/90 mmHg or the use of antihypertensive medications. HbA1c values were reported according to the National Glycohemoglobin Standardization Program [21].

### 2.5. Exposure and Outcomes

The primary exposure was the presence of GSECs at baseline kidney biopsy. The primary outcome was a composite endpoint defined as a ≥40% decline in eGFR from baseline or the initiation of kidney replacement therapy, including dialysis or kidney transplantation. A ≥40% decline in eGFR was defined based on the first documented measurement, irrespective of subsequent measurements. Longitudinal eGFR values during follow-up were obtained from electronic clinical records, and outcome events were assessed using all available laboratory and clinical data. Patients who did not reach the primary outcome were censored at the time of the last available clinical follow-up.

### 2.6. Statistical Analysis

Categorical variables are presented as percentages, and continuous variables as mean ± standard deviation or median (interquartile range), as appropriate based on their distribution. For regression analyses, age, baseline eGFR, and urinary protein excretion were analyzed as continuous variables; IFTA, medullary fibrosis, and medullary cast formation as ordinal variables; and sex, hypertension, and diabetes as binary variables. Group comparisons were performed using the chi-squared test for categorical variables and Student’s *t*-test or the Mann–Whitney U test for continuous variables. Binary logistic regression analysis was performed to identify factors associated with GSEC positivity. Kidney survival was assessed using the Kaplan–Meier method, and differences between groups were evaluated using the log-rank test. Patients were censored at the last follow-up visit or 31 October 2024, whichever came first. Cox proportional hazards models were used to estimate hazard ratios (HRs) and 95% confidence intervals (CIs). Multivariable models were constructed by adjusting for clinically relevant covariates selected a priori based on the prior literature, including age, sex, baseline eGFR, urinary protein excretion, IFTA, and underlying disease categories. There were no missing data for variables included in the regression analyses. All Cox models included 501 patients, among whom 112 experienced the primary outcome. The proportional hazards assumption was assessed using log-minus-log survival plots and was not violated. All statistical analyses were conducted using JMP version 17.0.0 (SAS Institute Inc., Cary, NC, USA). All tests were two-sided, and a *p* value < 0.05 was considered statistically significant.

## 3. Results

### 3.1. Patient Characteristics at the Time of Kidney Biopsy

Among 1165 patients who underwent native kidney biopsy at our institution, 501 met the inclusion criteria and were enrolled in the final analysis (Figure 1). Baseline clinical characteristics and primary clinicopathological diagnoses are summarized in Table 1 and Tables S1 and S2. The study cohort represented a clinically heterogeneous population with a broad spectrum of kidney diseases and varying degrees of kidney dysfunction and proteinuria.

**Figure 2:**
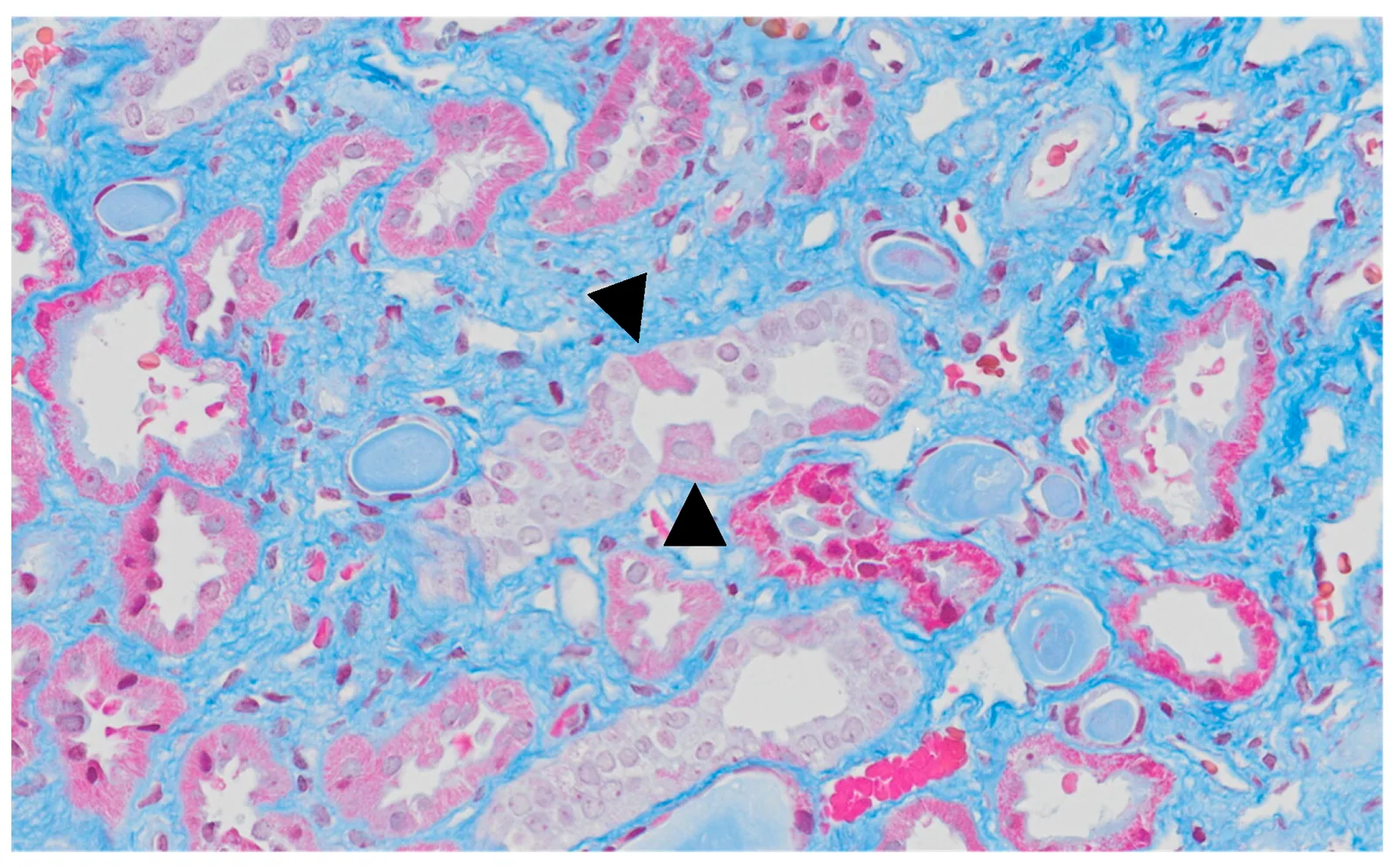
Representative histological images of GSECs. Markedly enlarged eosinophilic tubular epithelial cells with an approximately ≥1.5-fold increase in cell area compared with that of adjacent tubular epithelial cells (arrowheads). Original magnification, ×200.

The mean age of the cohort was 54.2 ± 17.8 years, and 222 patients (44.3%) were male. Diabetes and hypertension were present in 125 patients (25.0%) and 233 patients (46.5%), respectively. The mean baseline serum creatinine level was 1.06 ± 0.49 mg/dL, and the mean eGFR was 60.8 ± 26.3 mL/min/1.73 m^2^. Median urinary protein excretion was 1.1 g/gCr (interquartile range [IQR], 0.37–3.1). Hematuria was observed in 295 patients (58.9%), and urine casts were detected in 233 patients (46.5%). With respect to histopathological findings, mild to moderate IFTA was common, with IFTA grade 1 identified in 276 patients (55.1%) and grade 2 in 131 patients (26.1%). GSECs were identified in 94 patients (Figure 2) with evaluable medullary tissue, corresponding to approximately 18% of biopsies containing evaluable medulla.

### 3.2. Clinicopathological Associations of GSECs

Patients with GSEC positivity tended to be older and exhibited lower levels of proteinuria compared with those without GSECs. In contrast, no significant differences were observed in baseline eGFR between the two groups, suggesting that the presence of GSECs was not associated with baseline kidney function at the time of biopsy. These findings suggest that GSECs may reflect specific cellular or metabolic alterations rather than generalized kidney dysfunction.

**Figure 3:**
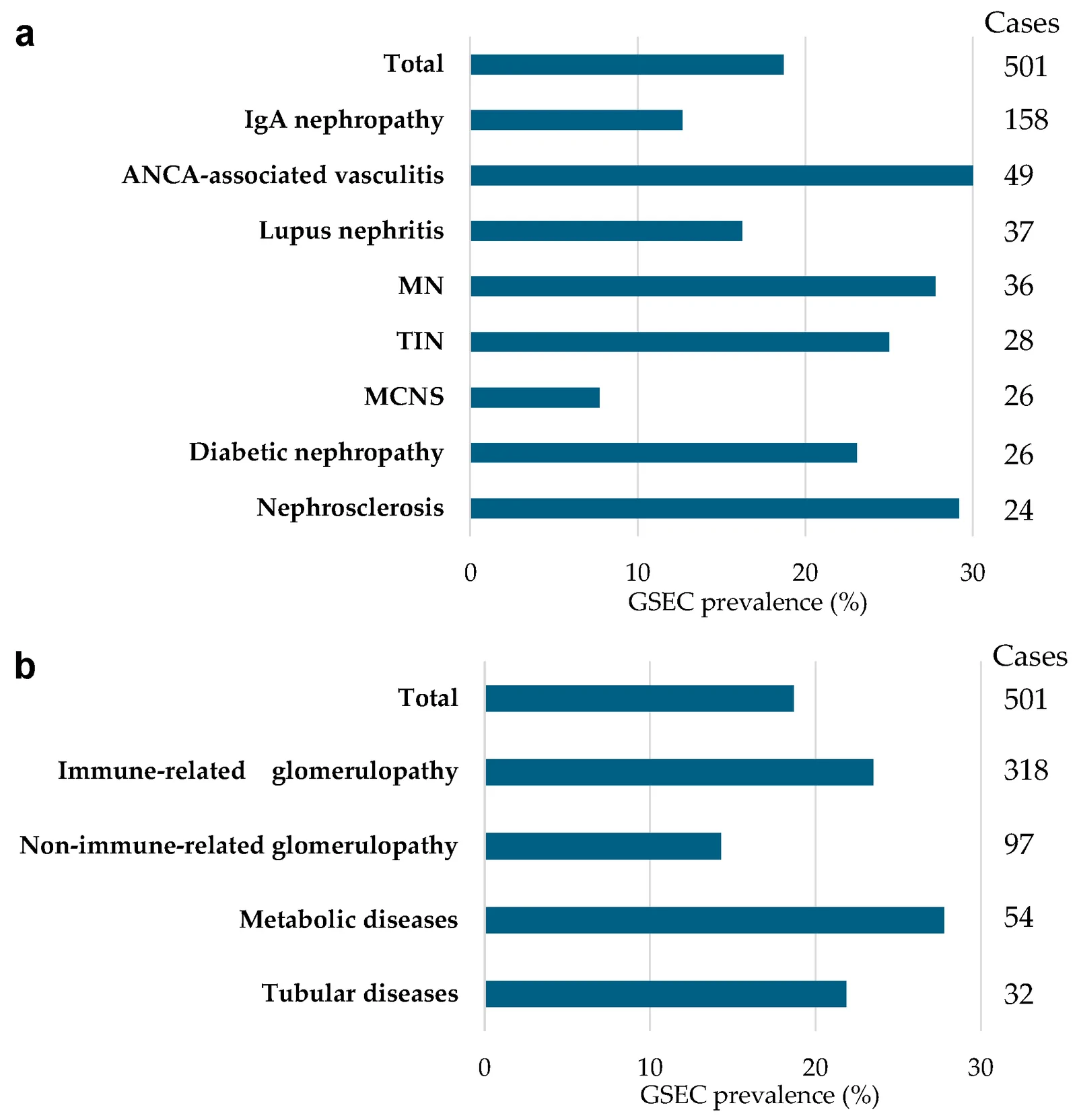
Prevalence of GSECs according to underlying kidney disease. (**a**) Analyses were restricted to underlying diseases with >20 cases. Differences among groups were assessed using the χ^2^ test (*p* = 0.039). ANCA, anti-neutrophil cytoplasmic antibody-associated vasculitis; lupus, MN, membranous nephropathy; TIN, tubulointerstitial nephritis; MCNS, minimal change nephrotic syndrome. (**b**) Underlying kidney diseases were categorized into immune-related, non-immune, metabolic, and tubulointerstitial diseases according to the classification shown in Table S2.

GSECs were identified across a broad spectrum of native kidney diseases, although their prevalence varied depending on the underlying pathological diagnosis. Among immune-mediated glomerular diseases, GSECs were most frequently observed in anti-neutrophil cytoplasmic antibody (ANCA)-associated vasculitis, where they were present in 30.0% of cases (Figure 3a). Relatively high frequencies were also observed in membranous nephropathy (27.8%), whereas the prevalence was lower in lupus nephritis (16%) and IgA nephropathy (13%). Among tubulointerstitial and vascular/metabolic disorders, GSECs were detected in 25% of tubulointerstitial nephritis cases, 29% of nephrosclerosis cases, and 23% of diabetic nephropathy cases. In contrast, minimal change nephrotic syndrome (MCNS) demonstrated a comparatively low prevalence of 7.7%. No patients with clinically diagnosed mitochondrial cytopathies were included in the present cohort. Despite these differences in prevalence, GSECs were observed across diverse pathological conditions involving immune-mediated, metabolic, vascular, and tubulointerstitial injury. To further evaluate disease category-specific distributions, underlying kidney diseases were additionally classified into immune-related, non-immune, metabolic, and tubulointerstitial categories (Figure 3b). GSECs were identified across all disease categories, with relatively higher frequencies in immune-related and metabolic/vascular disorders. These findings suggest that GSECs are not disease-specific lesions but rather nonspecific morphological alterations that may arise under various forms of tubular epithelial stress. Their broad distribution across unrelated disease entities further supports the interpretation that GSECs represent a shared cellular response rather than a disease-defining pathological feature.

To further clarify the clinicopathological correlates of GSECs, associations between GSEC positivity and clinically relevant variables were evaluated. The prevalence of GSECs tended to increase with advancing age, suggesting a potential association with aging-related cellular changes or cumulative metabolic stress within tubular epithelial cells. Consistent with this observation, univariate analyses demonstrated a trend toward an association between older age and GSEC positivity (Z = 1.93, *p* = 0.05; Table S3). In multivariable binary logistic regression analysis, age was independently associated with GSEC positivity (Table 2), with each 5-year increase in age associated with higher odds of GSEC presence (odds ratio [OR], 1.14; 95% confidence interval [CI], 1.04–1.24; *p* = 0.003). There was a tendency for GSECs to be more frequently observed in patients with lower levels of proteinuria, although urinary protein excretion was not independently associated with GSEC positivity in multivariable analysis (OR, 0.96; 95%CI, 0.88–1.03; *p* = 0.26). Similarly, baseline kidney function assessed by eGFR showed no significant association with GSECs (OR, 1.06 per 10 mL/min/1.73 m^2^ increase; 95%CI, 0.93–1.21; *p* = 0.35), indicating that GSECs were not linked to the severity of kidney dysfunction at the time of biopsy. No significant associations were identified between GSEC positivity and sex, hypertension, diabetes, or histopathological markers of chronic kidney injury, including IFTA, medullary fibrosis, and medullary cast formation. These findings suggest that GSECs are morphologically and clinically distinct from conventional chronic tubulointerstitial lesions associated with progressive nephron loss and fibrosis.

### 3.3. Prognostic Significance of GSECs

**Figure 4:**
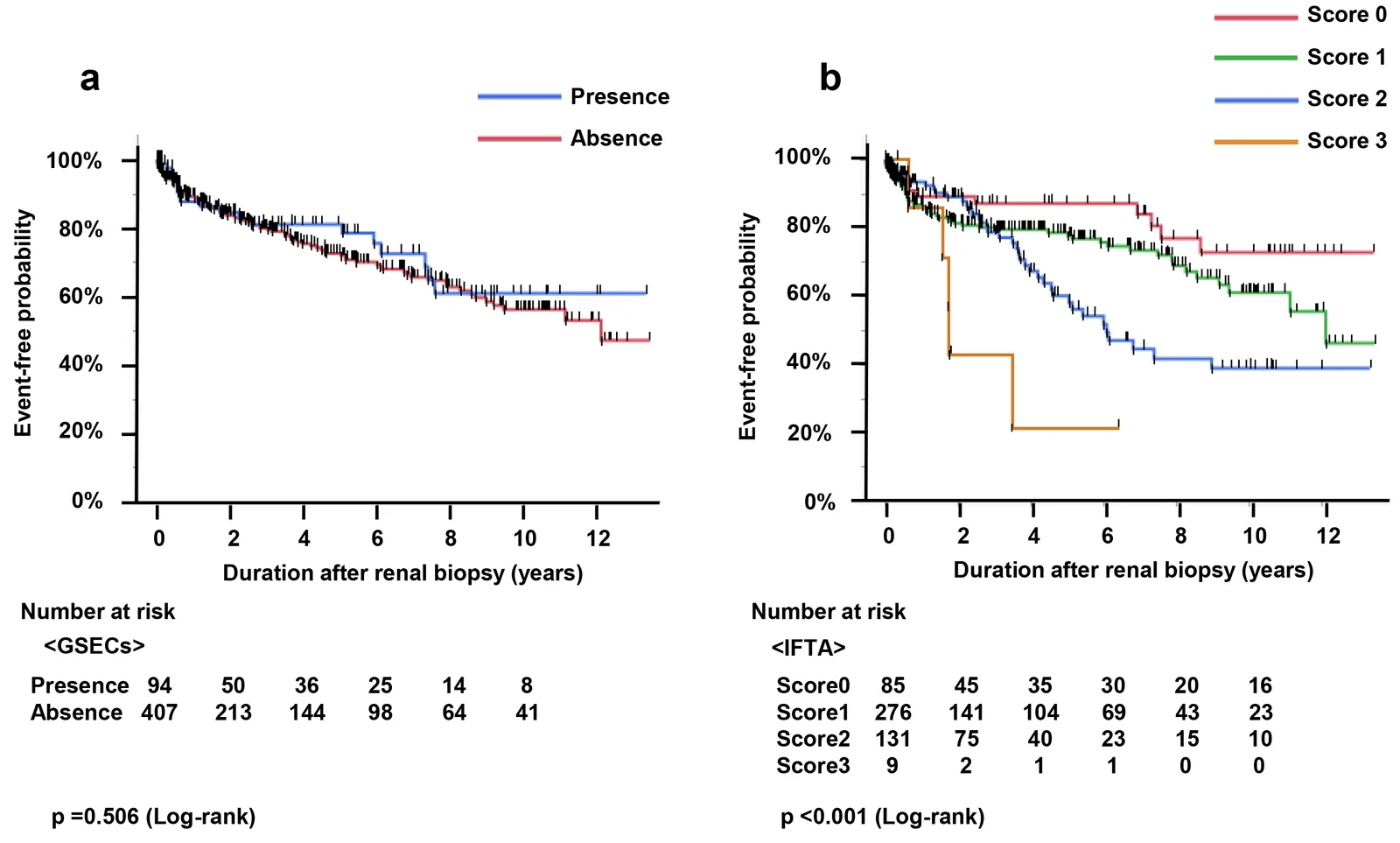
Kaplan–Meier curves for kidney outcomes according to GSEC status. (**a**) Renal survival rate stratified by GSECs. Log-rank test: *p* = 0.506. (**b**) Renal survival rate stratified by IFTA score. Log-rank test: *p* < 0.001. IFTA, interstitial fibrosis and tubular atrophy.

During a median follow-up period of 2.3 years (interquartile range, 0.7–5.0 years), 112 patients (22.3%) reached the composite kidney outcome, defined as a ≥40% decline in eGFR from baseline or the initiation of kidney replacement therapy. Kaplan–Meier survival analysis demonstrated that IFTA was significantly associated with poorer renal survival (Figure 4). Kidney survival progressively worsened according to increasing IFTA severity, and the Kaplan–Meier curves showed clear separation among IFTA categories (log-rank *p* < 0.001), consistent with the established prognostic significance of chronic tubulointerstitial injury. In contrast, no significant difference in renal survival was observed between patients with and without GSECs. The Kaplan–Meier curves for GSEC-positive and GSEC-negative patients remained closely overlapped throughout the observation period, with no apparent divergence during follow-up (log-rank *p* = 0.506), indicating that the presence of GSECs did not meaningfully influence long-term kidney prognosis.

To further evaluate the prognostic significance of GSECs, Cox proportional hazards models were applied using sequential multivariable adjustment (Table 3). In unadjusted analyses, GSEC positivity was not significantly associated with kidney outcomes. Similarly, after adjustment for clinically relevant covariates including age, sex, baseline eGFR, urinary protein excretion, and underlying disease categories, GSEC positivity remained non-significant. Additional adjustment for IFTA did not materially alter the results. Across all models, hazard ratios for GSEC positivity remained close to unity, indicating the absence of a clinically meaningful association between GSECs and adverse kidney outcomes. In contrast, IFTA consistently remained a strong and independent predictor of kidney prognosis across multivariable models, supporting the internal validity of the cohort and analytical approach. These findings emphasize the distinction between GSECs and established prognostic lesions associated with irreversible nephron loss and progressive chronic kidney disease. Similar results were obtained in the secondary analysis using the stricter definition requiring eccentric nuclear localization (nuclear displacement), with no significant association between GSEC positivity and kidney outcomes.

Subgroup analysis was performed in patients with IgA nephropathy, the largest disease category in the cohort, to evaluate the prognostic significance of GSECs within a more homogeneous disease population. Kaplan–Meier analysis demonstrated no significant difference in renal survival between patients with and without GSECs (Figure S1), further supporting the lack of prognostic significance of GSECs across different pathological backgrounds. Additional multivariable Cox analysis was not performed in this subgroup because of the limited number of outcome events.

## 4. Discussion

In this retrospective cohort study of patients undergoing native kidney biopsy, we demonstrated that GSECs are present in a substantial proportion of cases and are associated with specific clinical characteristics, particularly older age. No independent association between GSECs and kidney outcomes was observed in the present cohort, even after adjustment for established risk factors. These findings suggest that GSECs do not represent a prognostic lesion but rather a context-dependent morphological alteration of tubular epithelial cells. From a diagnostic perspective, GSECs should not be overinterpreted as disease-specific lesions or direct indicators of adverse kidney prognosis in routine native kidney biopsy evaluation. Because GSECs were identified across diverse pathological conditions, including immune-mediated, metabolic, vascular, and tubulointerstitial diseases, their presence alone appears insufficient to support a specific pathological diagnosis. Instead, GSECs may represent a nonspecific morphological reaction pattern associated with metabolic or cellular stress within the renal medulla. Recognition of this feature may nevertheless be diagnostically important to avoid confusion with other tubular alterations or overestimation of chronic tubular injury during pathological assessment. These findings raise the possibility that factors other than generalized tubular injury or aging-related changes may contribute to the characteristic phenotype previously described in inherited mitochondrial disorders.

GSECs have been primarily described in mitochondrial cytopathies and more recently in kidney allografts [15,17]. In mitochondrial diseases, GSECs are frequently observed as a prominent histological feature [17], whereas in kidney allografts, they have been linked to aging-related changes and chronic injury [15]. In the present study, we extend these observations by demonstrating that GSECs are also detectable in native kidney diseases, with a prevalence of 18%, which is comparable to or slightly higher than previously reported in transplant settings [15]. Furthermore, the variable distribution of GSECs across disease categories indicates that they are not specific to a particular disease entity but rather occur across a broad spectrum of kidney disorders. Importantly, despite their association with clinical characteristics, GSECs were not independently associated with kidney outcomes in this cohort. This contrasts with other histopathological findings such as interstitial fibrosis, tubular atrophy, and medullary cast formation, which have been consistently linked to adverse kidney prognosis, suggesting that GSECs represent a distinct type of histological finding that is not directly involved in progressive structural damage or nephron loss.

One of the most intriguing findings of this study was the marked variation in GSEC prevalence across different kidney diseases. In particular, GSECs were frequently observed in membranous nephropathy and ANCA-associated vasculitis but were uncommon in MCNS despite the presence of nephrotic-range proteinuria in both diseases. Several factors may account for these differences. First, differences in patient age may partly account for these findings, as patients with membranous nephropathy and ANCA-associated vasculitis were generally older than those with MCNS, and age was independently associated with GSEC positivity in the present study. Second, disease-specific biological mechanisms may also contribute. Membranous nephropathy is characterized by complement-mediated podocyte injury, whereas ANCA-associated vasculitis is associated with persistent inflammatory injury. In contrast, MCNS is primarily a podocytopathy with relatively limited tubulointerstitial injury and inflammation. Recent evidence also suggests that anti-nephrin autoantibodies contribute to the pathogenesis of a subset of patients with MCNS [22]. Therefore, the relatively low prevalence of GSECs in MCNS despite its immune-mediated nature suggests that GSEC formation is unlikely to reflect immune-mediated podocyte injury alone but instead may depend on downstream tubular metabolic responses or other disease-specific mechanisms. Finally, although both membranous nephropathy and MCNS often present with heavy proteinuria, differences in the composition and tubular handling of filtered proteins rather than the quantity of proteinuria itself may influence tubular metabolic stress and GSEC formation. In addition, ANCA-associated vasculitis and lupus nephritis involve marked innate immune activation, including neutrophil extracellular trap (NET) formation (NETosis) [23], raising the possibility that NETosis-related inflammatory pathways may contribute to tubular metabolic stress and GSEC formation. Although this hypothesis could not be addressed in the present study, it warrants future investigation. Collectively, these observations suggest that GSEC formation is unlikely to be determined solely by proteinuria but rather reflects the combined influence of aging, disease-specific biological mechanisms, and tubular metabolic adaptation. Accordingly, although GSECs were not independently associated with kidney prognosis, their distinct distribution among kidney diseases suggests potential biological relevance that warrants further mechanistic investigation.

The association between GSECs and mitochondrial alterations warrants further consideration. In mitochondrial cytopathies, tubular epithelial cells frequently exhibit cytoplasmic granularity, which has been associated with increased mitochondrial content and abnormal organelle morphology [24,25,26]. Similar features have also been described in aging kidneys and under conditions of chronic metabolic stress [27,28]. In this context, GSECs observed in native kidney disease may represent a convergent morphological phenotype potentially associated with metabolic or mitochondrial stress rather than a disease-specific lesion. Importantly, the renal medulla is particularly susceptible to hypoxia and metabolic stress due to its unique microenvironment characterized by low oxygen tension and high energy demand [12,29,30]. In addition to hypoxia, the renal medulla is exposed to marked osmotic stress and fluctuating oxygen availability, creating a unique metabolic microenvironment [31,32]. Tubular epithelial cells in this region require substantial mitochondrial activity to maintain ion transport and osmotic homeostasis [28]. Such conditions may render medullary tubular cells susceptible to cytoplasmic alterations under pathological stress conditions. However, because ultrastructural, molecular, and functional analyses were not performed in the present study, these mechanistic interpretations remain speculative. Furthermore, because GSECs may resemble enlarged eosinophilic tubular epithelial cells or reactive degenerative changes, awareness of their morphological characteristics may improve diagnostic reproducibility among pathologists and nephrologists.

Another important consideration is that GSEC-like changes may also be observed in histologically unremarkable or minimally altered kidney tissue. This suggests that GSECs do not necessarily indicate pathological injury but may instead represent a spectrum of physiological or adaptive responses to environmental or metabolic conditions. The presence of GSECs in both diseased and relatively preserved kidney tissue supports the notion that they reflect a context-dependent cellular state rather than a marker of irreversible damage. The lack of association between GSECs and kidney outcomes may be explained by their potential reversibility and limited contribution to structural damage. Unlike established prognostic markers such as IFTA, which reflect cumulative and often irreversible injury, GSECs may represent a transient cellular phenotype that does not directly lead to nephron loss. It is also conceivable that GSECs arise as part of an adaptive response aimed at maintaining cellular homeostasis under stress conditions, thereby reflecting a compensatory rather than maladaptive process. From a practical standpoint, our findings have implications for routine diagnostic pathology. Given that GSECs may be encountered across a wide range of kidney diseases and are not associated with clinical outcomes, overinterpretation of this feature might be avoided. In particular, distinguishing GSECs from other pathological findings with established prognostic value is essential to prevent misclassification of disease severity. Future studies incorporating quantitative assessment and molecular characterization may further clarify whether specific subtypes of GSECs carry distinct biological or clinical significance.

Several limitations should be acknowledged. First, this was a single-center retrospective study, and selection bias cannot be excluded. In particular, because only patients who underwent kidney biopsy and had evaluable medullary tissue were included, the study population may not fully represent the broader spectrum of CKD encountered in routine clinical practice. In addition, the relatively limited sample size within individual disease categories may have reduced statistical power for disease-specific analyses. Second, the study cohort included a heterogeneous range of underlying kidney diseases with distinct pathophysiological mechanisms, clinical courses, and prognostic determinants. Although this broad inclusion enabled evaluation of GSECs across diverse kidney pathologies, it may also have obscured disease-specific associations between GSECs and kidney outcomes. While subgroup analyses, including analyses restricted to IgA nephropathy, demonstrated findings generally consistent with those of the overall cohort, prognostic implications may differ according to the underlying disease entity. Therefore, larger disease-specific studies will be necessary to determine whether GSECs carry distinct biological or prognostic significance in particular pathological contexts. Third, the definition of GSECs relied on morphological assessment, which may introduce interobserver variability. In particular, distinguishing GSECs from enlarged tubular epithelial cells or intercalated cells may occasionally be challenging in routine histopathological evaluation. Although the qualitative morphological features of GSECs have been described in a previous study [17], no internationally standardized quantitative diagnostic criteria currently exist. Previous morphometric analysis used thresholds such as tubular epithelial cell enlargement exceeding two-fold relative to adjacent tubular cells [17]; however, these criteria have not been universally validated or incorporated into consensus pathological classifications. Therefore, we adopted provisional operational criteria based on previously reported morphological characteristics and internal consensus-based assessment. Nevertheless, some degree of subjective interpretation remains unavoidable, and the quantitative thresholds used in this study, including the ≥1.5-fold increase in tubular epithelial cell area, should be interpreted as exploratory rather than universally validated definitions. Furthermore, cases were classified as GSEC-positive when at least one morphologically identifiable GSEC was detected within evaluable medullary tissue. Because the amount of medullary tissue varied among biopsy specimens, sampling bias could not be completely excluded. Moreover, quantitative assessment of medullary tissue amount and pre-consensus interobserver agreement were not routinely recorded in this retrospective study and therefore could not be formally evaluated. Quantitative or semi-quantitative assessment of GSEC burden was also not systematically performed. Future studies incorporating standardized medullary sampling and quantitative assessment methods will be necessary to establish reproducible diagnostic criteria and improve inter-study comparability and pathological consistency. Fourth, mechanistic insights into mitochondrial function, tubular metabolism, or hypoxia-related pathways were not directly assessed in the present study. Because the interpretation of GSECs as manifestations of metabolic or mitochondrial stress remains largely speculative, future studies incorporating ultrastructural analyses, immunohistochemistry, transcriptomic approaches, or molecular characterization may provide further insight into the biological basis and functional significance of GSECs. In addition, patients with clinically confirmed mitochondrial cytopathies were not included in the present cohort. Therefore, direct comparisons between GSECs observed in native kidney diseases and those associated with inherited mitochondrial disorders could not be performed. Finally, the median follow-up duration was relatively modest, and longer-term observation may be necessary to fully exclude subtle associations between GSECs and kidney outcomes. Additional multicenter prospective studies with larger cohorts and disease-specific stratification will therefore be important to validate and extend the present findings.

## 5. Conclusions

GSECs are broadly distributed across diverse native kidney diseases and are not specific to individual disease entities. Nevertheless, the disease-dependent differences in their prevalence observed in this study suggest that GSECs may reflect distinct biological processes operating in different kidney diseases. Although GSECs were not independently associated with kidney outcomes in the present cohort, their characteristic distribution indicates potential biological relevance that deserves further mechanistic investigation. Further studies incorporating molecular and ultrastructural approaches are warranted to clarify the mechanisms underlying GSEC formation and its biological and diagnostic relevance.

## Figures and Tables

**Table 1:** Baseline clinical and histopathological characteristics according to GSEC status.

		All Patients (*n* = 501)	GSECs		*p*-Value
			Present (*n* = 94)	Absent (*n* = 407)	
Men		222 (44.3%)	45 (47.9%)	177 (43.5%)	0.49
Age, years		54.2 ± 17.8	57.5 ± 16.8	53.5 ± 18.0	0.05
BMI, kg/m^2^		22.7 ± 4.0	22.5 ± 3.9	22.7 ± 4.0	0.56
sBP, mmHg		127.4 ± 18.6	127.9 ± 18.3	127.3 ± 18.7	0.85
dBP, mmHg		79.4 ± 12.5	77.6 ± 11.1	79.8 ± 12.8	0.14
sCr, mg/dL		1.06 ± 0.49	1.03 ± 0.48	1.07 ± 0.50	0.50
eGFR, ml/min/1.73 m^2^		60.8 ± 26.3	61.4 ± 25.2	60.6 ± 26.6	0.72
HbA1c, %		5.7 (5.4–6.1)	5.7 (5.4–6.3)	5.7 (5.4–6.1)	0.66
U-P, g/gCr		1.1 (0.37–3.1)	0.89 (0.36–1.8)	1.2 (0.39–3.3)	0.30
Hematuria, %		295 (58.9%)	52 (55.3%)	243 (59.7%)	0.49
Urine pH		6.19 ± 0.81	6.13 ± 0.75	6.21 ± 0.82	0.45
Urine cast		233 (46.5%)	41 (43.6%)	192 (47.2%)	0.57
Hypertension		233 (46.5%)	40 (42.6%)	193 (47.4%)	0.42
DM		125 (25.0%)	25 (26.6%)	100 (24.6%)	0.69
ACE-I or ARB		173 (34.5%)	26 (27.7%)	147 (36.1%)	0.15
Ca blocker		175 (34.9%)	25 (26.6%)	150 (36.9%)	0.07
Statins		121 (24.2%)	14 (14.9%)	107 (26.3%)	0.06
IFTA	0	85 (17.0%)	14 (14.9%)	71 (17.4%)	0.32
	1	276 (55.1%)	60 (63.8%)	216 (53.1%)	
	2	131 (26.1%)	20 (21.3%)	111 (27.3%)	
	3	9 (1.8%)	0 (0.0%)	9 (2.2%)	
Medullary fibrosis	0	137 (27.3%)	23 (24.5%)	114 (28.0%)	0.89
	1	247 (49.3%)	49 (52.1%)	198 (48.6%)	
	2	117 (23.4%)	22 (23.4%)	95 (23.3%)	
Medullary inflammatory cell infiltration	0	366 (73.1%)	71 (75.5%)	295 (72.5%)	0.22
	1	111 (22.2%)	22 (23.4%)	89 (21.9%)	
	2	24 (4.8%)	1 (1.1%)	23 (5.7%)	
Medullary cast formation	0	259 (51.7%)	50 (53.2%)	209 (51.4%)	0.54
	1	176 (35.1%)	34 (36.2%)	142 (34.9%)	
	2	66 (13.2%)	10 (10.6%)	56 (13.8%)	

Values reported as *n* (%) for categorical variables and mean ± standard deviation or median (interquartile range) for continuous variables. BMI, body mass index; sBP, systolic blood pressure; dBP, diastolic blood pressure; sCr, serum creatinine; eGFR, estimated glomerular filtration rate; HbA1c, hemoglobin A1c; U-P, urinary protein excretion; DM, diabetes mellitus; ACE-I, angiotensin-converting enzyme inhibitor; ARB, angiotensin II type 1 receptor blocker; IFTA, interstitial fibrosis and tubular atrophy. GSECs were coded as 0 (absent) and 1 (present).

**Table 2:** Binary logistic regression analysis for factors associated with GSEC.

	GSECs (Present vs. Absent)		
	Estimated Odds	95%CI	*p*-Value
Sex (male)	1.20	0.75–1.91	0.44
Age (increased by 5 years)	1.14	1.04–1.24	0.003 **
eGFR (increased by 10 mL/min/1.73 m^2^)	1.06	0.93–1.21	0.35
U-P	0.96	0.88–1.03	0.26
Hypertension	0.67	0.40–1.10	0.12
Diabetes	1.00	0.58–1.71	0.99
IFTA	0.85	0.54–1.33	0.48
Medullary fibrosis	1.07	0.72–1.57	0.75
Medullary cast formation	0.90	0.59–1.34	0.59

95%CI, 95% confidence interval; eGFR, estimated glomerular filtration rate; U-P, urinary protein excretion; IFTA, interstitial fibrosis and tubular atrophy. ** *p* < 0.01.

**Table 3:** Multivariable Cox proportional hazards models for kidney outcomes.

	Model 0 HR (95%CI)	Model 1 HR (95%CI)	Model 2 HR (95%CI)	Model 3 HR (95%CI)	Model 4 HR (95%CI)
GSECs (present vs. absent)	0.85 (0.52–1.39)	0.74 (0.45–1.22)	0.75 (0.46–1.24)	0.75 (0.45–1.23)	0.78 (0.48–1.29)

Model 0: No adjustment factors. Model 1: Sex, Age. Model 2: Model 1 + Urinary protein, eGFR. Model 3: Model 2 + IFTA. Model 4: Model 3 + underlying disease categories (immune-related glomerulopathy, non-immune-related glomerulopathy, metabolic diseases, and tubular diseases). HR, hazard ratio; 95%CI, 95% confidence interval; IFTA, interstitial fibrosis and tubular atrophy.

## Data Availability

The data presented in this study are available on request from the corresponding author. The data are not publicly available due to privacy and ethical restrictions.

## Supplementary Materials

The following supporting information can be downloaded at: https://www.mdpi.com/article/10.3390/diagnostics16172822/s1, Figure S1: Renal survival rate stratified by the presence of GSEC and IFTA score in IgA nephropathy; Table S1: Clinical and histopathological findings for all patients and for patients stratified by IFTA score; Table S2: Clinicopathologic diagnoses of the study population; Table S3: Univariate analyses of variables associated with GSEC presence; File S1. STROBE Statement.

## Abbreviations

The following abbreviations are used in this manuscript:

GSECs	Granular swollen epithelial cells
IFTA	Interstitial fibrosis and tubular atrophy
eGFR	estimated glomerular filtration rate
BMI	body mass index
HbA1c	hemoglobin A1c
ACE-Is	angiotensin-converting enzyme inhibitors
ARBs	angiotensin II receptor blockers
MCNS	minimal change nephrotic syndrome
