# Supplementary material for: Granular Swollen Epithelial Cells in Native Kidney Biopsies: Prevalence, Clinicopathological Associations and Kidney Outcomes
Source: Diagnostics (Basel). 2026 Sep 2;16(17):2822. doi: 10.3390/diagnostics16172822 (PMC13565308; doi:10.3390/diagnostics16172822)
Supplement: Supplementary file 1 [file diagnostics-16-02822-s001.zip › diagnostics-4344968-supplementary.pdf]

Supplemental Material

Table of contents

1. Supplemental Table

Table S1. Clinical and histopathological findings for all patients and for patients stratified by IFTA score.

Table S2. Clinicopathologic diagnoses of the study population.

Table S3. Univariate analyses of variables associated with GSEC presence.

2. Supplemental Figure

Figure S1. Renal survival rate stratified by the presence of GSEC and IFTA score in IgA nephropathy.

3. Supplemental File

File S1. STROBE Statement.

Table S1. Clinical and histopathological findings for all patients and for patients stratified by IFTA score.

		All patients (n=501)	IFTA score				p for trend
			0 (n=85)	1 (n=276)	2 (n=131)	3 (n=9)	
Men		222 (44.3%)	37 (43.5%)	128 (46.4%)	52 (39.7%)	5 (55.6%)	0.56
Age, years		54.2±17.8	41.6±18.0	54.9±17.1	60.8±14.9	55.6±18.0	<0.001
BMI, kg/m^2		22.7±4.0	22.9±4.2	22.7±3.6	22.4±4.5	23.5±3.9	0.61
sBP, mmHg		127.4±18.6	120.4±15.8	127.1±18.9	132.0±18.4	135.7±17.9	<0.001
dBP, mmHg		79.4±12.5	77.5±10.5	79.6±12.1	80.4±14.1	75.9±15.6	0.26
sCr, mg/dL		1.06±0.49	0.78±0.27	0.96±0.40	1.41±0.55	1.90±0.50	<0.001
eGFR, ml/min/1.73 m^2		60.8±26.3	84.6±26.5	64.0±22.2	40.8±17.1	28.9±7.66	<0.001
HbA1c, %		5.7 (5.4-6.1)	5.5 (5.3-5.8)	5.8 (5.5-6.2)	5.8 (5.4-6.1)	6.1 (5.1-6.9)	0.007
U-P, g/gCr		1.1 (0.37-3.1)	0.70 (0.16-3.50)	1.01 (0.38-2.88)	1.30 (0.57-2.84)	4.99 (2.74-5.71)	0.005
Hematuria, %		295 (58.9%)	44 (51.8%)	177 (64.1%)	67 (51.1%)	7 (77.8%)	0.72
Urine pH		6.19±0.81	6.59±0.91	6.20±0.77	5.92±0.73	6.22±0.62	<0.001
Urine cast		233 (46.5%)	27 (31.8%)	132 (47.8%)	68 (51.9%)	6 (66.7%)	0.004
Hypertension		233 (46.5%)	21 (24.7%)	123 (44.6%)	84 (64.1%)	5 (55.6%)	<0.001
DM		125 (25.0%)	16 (18.8%)	68 (24.6%)	36 (27.5%)	5 (55.6%)	0.06
ACE-I or ARB		173 (34.5%)	11 (12.9%)	102 (37.0%)	57 (43.5%)	3 (33.3%)	<0.001
Ca blocker		175 (34.9%)	16 (18.8%)	86 (31.2%)	67 (51.1%)	6 (66.7%)	<0.001
Statins		121 (24.2%)	16 (18.8%)	71 (25.7%)	31 (23.7%)	3 (33.3%)	0.58
Medullary fibrosis	0	137 (27.3%)	63 (74.1%)	69 (25.0%)	5 (3.8%)	0 (0.0%)	<0.001
	1	247 (49.3%)	20 (23.5%)	163 (59.1%)	63 (48.1%)	1 (11.1%)	
	2	117 (23.4%)	2 (2.4%)	44 (15.9%)	63 (48.1%)	8 (88.9%)	
Medullary inflammatory cell infiltration	0	366 (73.1%)	81 (95.3%)	220 (79.7%)	63 (48.1%)	2 (22.2%)	<0.001
	1	111 (22.2%)	4 (4.7%)	54 (19.6%)	51 (38.9%)	2 (22.2%)	
	2	24 (4.8%)	0 (0.0%)	2 (0.7%)	17 (13.0%)	5 (55.6%)	
Medullary cast formation	0	259 (51.7%)	78 (91.8%)	153 (55.4%)	27 (20.6%)	1 (11.1%)	<0.001
	1	176 (35.1%)	7 (8.2%)	97 (35.1%)	70 (53.4%)	2 (22.2%)	
	2	66 (13.2%)	0 (0.0%)	26 (9.4%)	34 (26.0%)	6 (66.7%)	
GSECs	0	407 (81.2%)	71 (83.5%)	216 (78.3%)	111 (84.7%)	9 (100.0%)	0.36
	1	94 (18.7%)	14 (16.5%)	60 (21.7%)	20 (15.3%)	0 (0.0%)	

Values reported as n (%) for categorical variables and mean ± standard deviation or median (interquartile range) for continuous variables. BMI, body mass index; sBP, systolic blood pressure; dBP, diastolic blood pressure; sCr, serum creatinine; eGFR, estimated glomerular filtration rate; HbA1c, hemoglobin A1c; U-P, urinary protein excretion; DM, diabetes mellitus; ACE-I, angiotensin–converting enzyme inhibitor; ARB, angiotensin II type 1 receptor blocker; IFTA, interstitial fibrosis and tubular atrophy.

Table S2. Clinicopathologic diagnoses of the study population.

Immune-related glomerulopathy (n=318)	Non-immune-related glomerulopathy (n=97)	Metabolic diseases (n=54)	Tubular diseases (n=32)
IgA nephropathy (158)	MCNS (26)	Diabetic nephropathy (26)	TIN (28)
ANCA-associated vasculitis (49)	Minor glomerular changes (20)	Nephrosclerosis (24)	BK virus–associated nephropathy (1)
Lupus nephritis (37)	FSGS (18)	Obesity-related nephropathy (4)	Medullary cystic kidney disease (1)
MN (36)	TBM (14)		Acute tubular necrosis (1)
IgA vasculitis (16)	Amyloidosis (8)		Light-chain proximal tubulopathy (1)
MGPN (11)	TMA (4)		
IRGN (3)	Alport syndrome (4)		
Cryoglobulinemic vasculitis (3)	Fabry disease (2)		
Immunotactoid nephropathy (2)	Lipoprotein glomerulopathy (1)		
Non-IgA mesangial proliferative glomerulonephritis (1)			
Anti-GBM glomerulonephritis (1)			
Polyarteritis nodosa (1)			

ANCA, anti-neutrophil cytoplasmic antibody; MN, membranous nephropathy; MPGN, membranoproliferative glomerulonephritis; IRGN, infection-related glomerulonephritis; GBM, glomerular basement membrane; MCNS, minimal change nephrotic syndrome; FSGS, focal segmental glomerulosclerosis; TBM, thin basement membrane disease; TMA, thrombotic microangiopathy; TIN, tubulointerstitial nephritis. TIN includes sarcoid nephropathy and IgG4-related kidney disease. TMA includes calcineurin inhibitor-associated nephropathy.

Table S3. Univariate analyses of variables associated with GSEC presence.

	Test	Z statistic	p-value
Age	Wilcoxon	1.93	0.05
BMI	Wilcoxon	-0.58	0.56
sBP	Wilcoxon	-0.20	0.85
dBP	Wilcoxon	-1.46	0.14
eGFR	Wilcoxon	0.36	0.72
HbA1c	Wilcoxon	0.44	0.66
U-P	Wilcoxon	-1.04	0.30
IFTA	Jonckheere-Terpstra	-0.92	0.36
Medullary fibrosis	Jonckheere-Terpstra	0.26	0.79
Medullary inflammatory cell infiltration	Jonckheere-Terpstra	-0.81	0.42
Medullary cast formation	Jonckheere-Terpstra	-0.52	0.60

Wilcoxon rank-sum test was used for continuous variables, and Jonckheere-Terpstra test was used for ordinal variables. BMI, body mass index; sBP, systolic blood pressure; dBP, diastolic blood pressure; eGFR, estimated glomerular filtration rate; HbA1c, hemoglobin A1c; U-P, urinary protein excretion; IFTA, interstitial fibrosis and tubular atrophy.

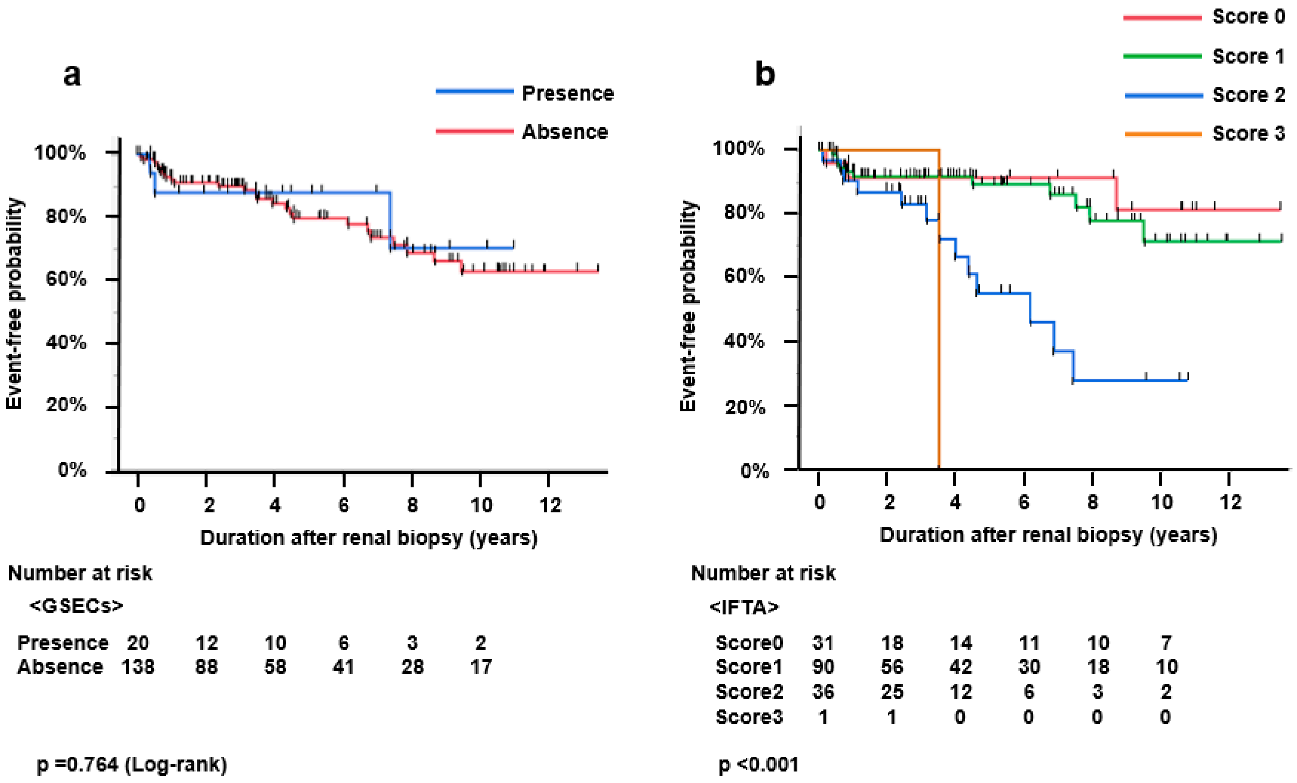

Figure S1. Renal survival rate stratified by the presence of GSEC and IFTA score in IgA nephropathy.

(a) Renal survival rate stratified by the presence of GSEC. Log-rank test: p = 0.764. (b) Renal survival rate stratified by IFTA score. Log-rank test: p <0.001. IFTA, interstitial fibrosis and tubular atrophy.

File S1. STROBE Statement

	Item No	Recommendation	Page No
**Title and abstract**	1	(*a*) Indicate the study's design with a commonly used term in the title or the abstract	1
		(*b*) Provide in the abstract an informative and balanced summary of what was done and what was found	1
**Introduction**			
Background/rationale	2	Explain the scientific background and rationale for the investigation being reported	2
Objectives	3	State specific objectives, including any prespecified hypotheses	2
**Methods**			
Study design	4	Present key elements of study design early in the paper	3
Setting	5	Describe the setting, locations, and relevant dates, including periods of recruitment, exposure, follow-up, and data collection	3
Participants	6	(*a*) Give the eligibility criteria, and the sources and methods of selection of participants. Describe methods of follow-up	3-4
		(*b*) For matched studies, give matching criteria and number of exposed and unexposed	N/A
Variables	7	Clearly define all outcomes, exposures, predictors, potential confounders, and effect modifiers. Give diagnostic criteria, if applicable	3-4
Data sources/ measurement	8*	For each variable of interest, give sources of data and details of methods of assessment (measurement). Describe comparability of assessment methods if there is more than one group	3-4
Bias	9	Describe any efforts to address potential sources of bias	3-4
Study size	10	Explain how the study size was arrived at	3

Quantitative variables	11	Explain how quantitative variables were handled in the analyses. If applicable, describe which groupings were chosen and why	4
Statistical methods	12	(*a*) Describe all statistical methods, including those used to control for confounding	4
		(*b*) Describe any methods used to examine subgroups and interactions	4, 9
		(*c*) Explain how missing data were addressed	N/A
		(*d*) If applicable, explain how loss to follow-up was addressed	N/A
		(*e*) Describe any sensitivity analyses	3, 7, 9
**Results**			
Participants	13*	(a) Report numbers of individuals at each stage of study—eg numbers potentially eligible, examined for eligibility, confirmed eligible, included in the study, completing follow-up, and analysed	3-5
		(b) Give reasons for non-participation at each stage	
		(c) Consider use of a flow diagram	Figure 1
Descriptive data	14*	(a) Give characteristics of study participants (eg demographic, clinical, social) and information on exposures and potential confounders	5-6, 8
		(b) Indicate number of participants with missing data for each variable of interest	
		(c) Summarise follow-up time (eg, average and total amount)	
Outcome data	15*	Report numbers of outcome events or summary measures over time	8

Main results	16	(*a*) Give unadjusted estimates and, if applicable, confounder-adjusted estimates and their precision (eg, 95% confidence interval). Make clear which confounders were adjusted for and why they were included<br>(*b*) Report category boundaries when continuous variables were categorized<br>(*c*) If relevant, consider translating estimates of relative risk into absolute risk for a meaningful time period	3-4, 7-9
Other analyses	17	Report other analyses done—eg analyses of subgroups and interactions, and sensitivity analyses	7, 9
**Discussion**			
Key results	18	Summarise key results with reference to study objectives	9
Limitations	19	Discuss limitations of the study, taking into account sources of potential bias or imprecision. Discuss both direction and magnitude of any potential bias	11
Interpretation	20	Give a cautious overall interpretation of results considering objectives, limitations, multiplicity of analyses, results from similar studies, and other relevant evidence	9-11
Generalisability	21	Discuss the generalisability (external validity) of the study results	11
**Other information**			
Funding	22	Give the source of funding and the role of the funders for the present study and, if applicable, for the original study on which the present article is based	12
